# Can Baseline Characteristics Predict Successful Outcomes after Individual, Physiotherapist-Led Rehabilitation in Patients with Chronic Musculoskeletal Pain?

**DOI:** 10.1155/2023/5182996

**Published:** 2023-06-16

**Authors:** Elisabeth Bondesson, Anna Jöud, Marcelo Rivano Fischer, Anna Trulsson Schouenborg

**Affiliations:** ^1^Lund University, Department of Clinical Sciences Lund, Lund, Sweden; ^2^Skåne University Hospital, Department of Neurosurgery and Pain Rehabilitation, Lund, Sweden; ^3^Lund University, Department of Laboratory Medicine, Division of Occupational and Environmental Medicine, Lund, Sweden; ^4^Skåne University Hospital, Department of Research and Education, Lund, Sweden; ^5^Lund University, Department of Health Sciences, Research Group Rehabilitation Medicine, Lund, Sweden; ^6^Lund University, Department of Health Sciences, Research Group Human Movement-Health and Rehabilitation, Lund, Sweden

## Abstract

**Background:**

No strong and consistent variables to predict outcome after pain rehabilitation have been reported in patients with chronic musculoskeletal pain. The aim of the present study was to clarify if baseline variables could predict successful outcome after a unique, individualized, physiotherapist-led rehabilitation of nine sessions.

**Methods:**

In 274 individuals with severe chronic musculoskeletal pain, the risk ratio (RR) and 95% confidence intervals (CIs) were estimated for potentially predictive baseline variables on successful outcomes of pain management, overall health, and pain rating.

**Results:**

Statistically significant results show that patients rating moderate or severe baseline pain were in both cases 14% less likely to improve pain management compared to patients rating mild baseline pain (RR = 0.86; 95% CI 0.77–0.97, RR = 0.86; 95% CI 0.74–1.00). Patients with the shortest pain duration were 1.61 times more likely to improve overall health (RR = 1.61; 95% CI 1.13–2.29) compared to patients reporting the longest pain duration (>5 years). Patients reporting anxiety/depression or severe pain were in both cases 1.48 times more likely to improve overall health compared to better baseline presentations (RR = 1.48; 95% CI 1.16–1.88, RR = 1.48; 95% CI 1.03–2.15). Patients with regional/generalized pain were 36% less likely to rate pain reduction (RR = 0.64; 95% CI 0.41–1.00) compared to patients rating localized baseline pain. Of 17 potentially predictive baseline variables, four reached statistical significance for at least one of the three outcomes; although none of them for all three outcomes.

**Conclusions:**

Of 17 potentially predictive baseline variables, mild pain ratings, short pain duration, and localized baseline pain were statistically significantly associated with improvements after individual, physiotherapist-led rehabilitation for patients with chronic musculoskeletal pain. This suggests that this type of rehabilitation probably should be offered early in the pain process. Reporting anxiety/depression or severe pain at the baseline did not hinder the improvements of overall health.

## 1. Introduction

Chronic musculoskeletal pain (CMP) is a persisting or reoccurring pain lasting more than three months, originating from musculoskeletal structures such as joints, bones, muscles, tendons, or from multiple body areas and/or components, such as regional or widespread pain [1–4]. About 20% of the European population suffers from chronic musculoskeletal pain. Chronic neck and/or lower back pain are often described as the leading cause of disability [5]. Further, chronic pain and consequences thereof can limit functions, activities, and participation in activities important in everyday life [6].

Recommended treatments for chronic pain are combinations of interdisciplinary interventions with psychological, educational, physical, and occupational components, often delivered in group format over weeks or months [7–10]. As yet, no consensus exists regarding the exact setting, content, or optimal treatment dosage [8, 10–12]. Additionally, current approaches stress the importance of providing individually tailored care for subgroups of patients [8, 13–15] and, perhaps more importantly, international guidelines [7]. Therefore, one-to-one physiotherapy approaches in patients with chronic pain have been advocated [16]. Such individual rehabilitation has been suggested to comprise the specific needs, preferences, and abilities of patients. Thereby somatic (including the pain mechanisms), psychosocial, motivational, and behavioral factors are considered [7, 17, 18]. To meet these requirements, a one-to-one rehabilitation program, the physiotherapy pain rehabilitation program (PT-PRP) was developed at a specialized pain rehabilitation unit [19]. The program was intended for patients with unresolved pain problems but still not in the need of interdisciplinary pain rehabilitation. Patients were usually referred from primary care.

Even if interdisciplinary interventions, as well as one-to-one physiotherapy approaches can be effective, not all patients benefit from these interventions. Therefore, it is important to identify the patients that benefit the most from different interventions [8]. An important question is what are the baseline factors that may predict successful outcomes from different interventions? As for multimodal pain rehabilitation, no strong and consistent variables to predict outcome after rehabilitation have been reported in systematic reviews [20–23]. To the best of our knowledge, systematic reviews concerning predictive factors for one-to-one physiotherapy in patients suffering from CMP are lacking. Therefore, there is a need to study this further.

The aim of the present study was to analyze if baseline variables could predict successful outcomes after individualized, one-to-one, physiotherapist-led rehabilitation for patients with CMP who were refractory to preceding similar treatments. The outcomes were measured as follows: (i) ability to manage overall life circumstances, (ii) ratings of perceived overall health status, and (iii) ratings of pain.

## 2. Materials and Methods

### 2.1. The Patient Cohort

Patients with CMP in the present study were consecutively included in the rehabilitation program between Jan 1st 2014 and 20th Nov 2018, according to a structured, written routine and according to regular clinical care. The patients had been referred to physiotherapy within a specialized pain unit in Sweden due to unresolved pain problems. Most patients were referred from primary care. In total, 486 consecutive patients were referred to the physiotherapy department and eligible for inclusion. The *inclusion criteria* were as follows: chronic (>3 months) musculoskeletal pain and 18 years of age or older. The *exclusion criteria* were as follows: acute psychiatric illness or acute crisis, urgent social or economic difficulties, present alcohol or drug abuse or psychological consequences deemed to hinder the improvements of physiotherapeutic interventions. The exclusion criteria were assessed by MD or a psychologist. The patient cohort has been described in detail in a previous study [19].

**Figure 1:**
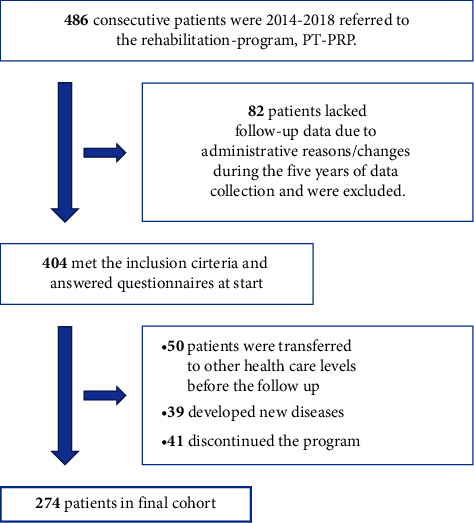
Flow chart of the inclusion and exclusion of the 486 patients referred to the Physiotherapy Pain Rehabilitation Program, PT-PRP, during the data collection period 2014–2018.

Out of the 486 eligible patients, not all were available to be included in the final clinical cohort. This was due to the following reasons:

- 82 patients lacked follow-up data due to administrative reasons/changes during the 5 years of data collection. For example, questionnaires were changed or exchanged
- 50 patients were transferred to other health care levels (for example, the multimodal rehabilitation program) before the follow-up
- 39 developed new diseases requiring specific medical care during the program
- 41 discontinued the program (see Figure 1).

In the final cohort, 274 patients (mean age 42 years, 71% women), patients with CMP (numeric pain rating scale (NPRS) median 7/10, pain duration median 2.8 years) and moderate disability, were included [19] (see Table 1).

### 2.2. Rehabilitation Program PT-PRP

**Figure 2:**
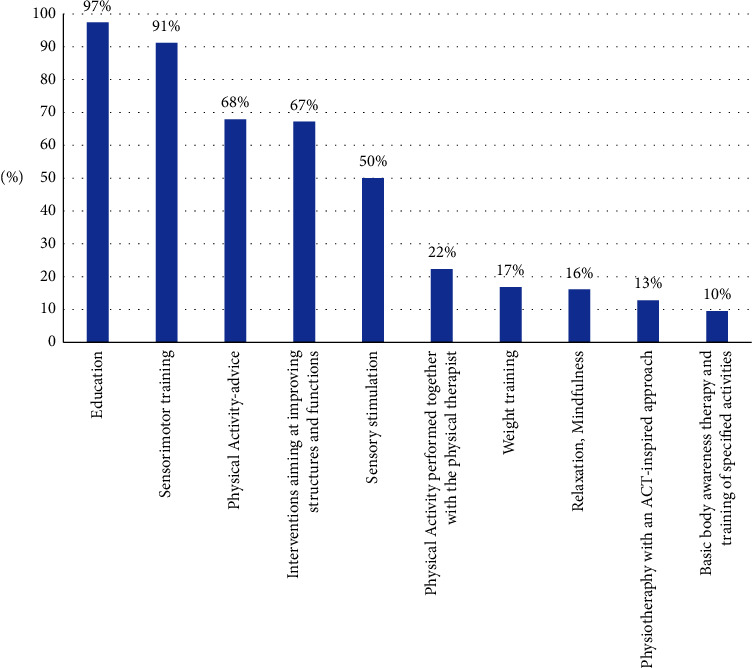
Percentages of the whole population, *n* = 274, that participated in the different interventions during the rehabilitation program PT-PRP.

In the PT-PRP, all patients received an initial evaluation consisting of a medical history, a physical examination, and questionnaires. Further, the PT-PRP consisted mainly of patient education, sensorimotor training, physical activity advice, and interventions aiming at improving structures and functions (see Figure 2). The most common combination of interventions was as follows: education, sensorimotor training, and interventions aiming at improving structures and functions and was practiced by 138 patients, 50%. The rehabilitation was combined with exercises and regimes at home. The treatment could also, based on individual needs, include for example sensory stimulation, weight training, and relaxation. The PT-PRP lasted a median of five months. During these five months, a median of nine physiotherapist-led sessions took place in the unit. The duration of the rehabilitation did not exceed six months. The PT-PRP is described in detail by Trulsson Schouenborg et al. [19].

### 2.3. Outcomes and Potentially Predictive Baseline Variables

Patients answered questionnaires prior to the rehabilitation, at discharge and one year after discharge, using patient reported outcome measures (PROM). The PROMs used in the PT-PRP, and hence in the present study, were chosen based on clinical experience and the literature [24–26]. The PROMs are recommended by both Initiative on Methods, Measurement, and Pain Assessment in Clinical Trials (IMMPACT) and validation and application of a patient relevant core outcome set to assess effectiveness of multimodal PAIN therapy (VAPAIN) as core outcomes in studies investigating chronic pain populations [26, 27].

The three main outcomes were as follows:

- One question on ability to manage overall life circumstances according to the Swedish Quality Registry for Pain Rehabilitation (SQRP) is as follows: “has your rehabilitation influenced your ability to manage overall life circumstances?,” 1–5, 1 = much worse, 2 = worse, 3 = no change, 4 = improved, and 5 = much improved. Successful outcome after rehabilitation was defined as rating “improved” or “much improved” at discharge [25].
- Ratings of perceived overall health status were evaluated with EQVAS from the EuroQol five-dimension scale (EQ-5D, a measurement of quality of life). Assessments of perceived health were rated on a scale from 0 = “worst health imaginable,” to 100 = “best health imaginable” [28]. Successful outcome after rehabilitation was defined as a minimal clinical important difference (MCID) of 20% as measured between start and discharge [28].
- Perceived pain during last week according to NPRS, 0–10, 0 corresponding to no pain, 10 corresponding to worst pain imaginable [29]. Successful rehabilitation was defined as an improvement in MCID of 2 points between start and discharge [30, 31].

The patients' ratings of the question on “ability to manage overall life circumstances,” pain, and perceived health status at discharge are presented in detail in a previous study by Trulsson Schouenborg [19]. In short, the results of the single question, “Has your rehabilitation influenced your ability to manage overall life circumstances?” showed that 85% rated “improved” or “much improved” at discharge. The patients' ratings of pain at discharge showed that 45% of the patients rated clinically important improvements on pain. Also, 50% of the patients rated improved perceived overall health status at discharge and the figures were similar at 1-year follow-up. Since there were no statistically significant differences in main outcomes from discharge to 1-year follow-up, the data at discharge were used in the present study, and due to that a larger number of patients had answered the questionnaires at discharge (Table 2).

Potentially predictive baseline variables including categorization are presented in Table 3. The following variables were categorized according to the descriptions below:

- NPRS was categorized into mild, moderate, and severe pain categories [36].
- DRI was categorized into three categories according to the tertiles of the DRI results in the present cohort [32, 33].
- EQVAS was categorized into three arbitrary categories (0–40, 41–70, and 71–100) based on our results of very few individual rating EQVAS 80–100 at the baseline.
- The single question, “What is your opinion on your ability to reduce your pain/ailment,” was aggregated into three categories.
- The questions on physical exercise, leisure exercise, and sedentary time were respectively divided into three categories.
- The single question on anxiety/depression in EQ-5D, 3L, and 5L was dichotomized into either “reporting NO anxiety/depression” (=answer no. 1 + 2 in EQ-5D 3L and in EQ-5D 5L) or into “reporting anxiety/depression” (=answer no. 3 in EQ-5D 3L and no 3 + 4 + 5 in EQ-5D 5L) [37].
- “Number of sessions during the rehabilitation-period” was categorized into three categories according to the tertiles in the present cohort and also since the literature shows unknown dosage [38].
- “Number of different types of interventions” was categorized according to the tertiles obtained in the present cohort.

Our reasons for using categories for pain duration and “ability to reduce pain” were that they were categorized already in the questionnaire. BMI and NPRS have clinically meaningful categories, making it easier to interpret in the clinical situation. Variables of age, DRI, and EQVAS could have been included as continuous variables; however, due tothe relatively small study sample, we made the decision to categorize them to increase power.

### 2.4. Ethical Approval and Informed Consent Statement to Participate

Data in the present study were retrieved from a previous study, and all patients were subject to “usual care” [19]. Informed consent from the participants was obtained before start of the study. The study was approved by Swedish Ethical Review Agency, Dnr: 2019–03701 and in accordance with “Declaration of Helsinki,” and all procedures were performed in accordance with relevant guidelines.

### 2.5. Data Management and Statistical Analyses

Descriptive statistics were used to analyze frequencies and distribution, and in measurements considered to be ordinal scale data, median, minimum–maximum, and quartiles were used. In analyzes of within-group comparisons, nonparametric statistics were used to test for differences (Wilcoxon signed rank test). To calculate the intercorrelation between main outcomes, nonparametric bivariate correlation and Spearman's correlation coefficient (*r*_s_) were used.

The risk ratio (RR) was calculated using modified Poisson regression, i.e., Poisson regression with a robust error variance, using the PROC GENMOD procedure and the repeated subject statement [39]. In a first step, univariate modified Poisson regression modelling was used to estimate the RR and 95% confidence intervals (CIs) for each of the three outcomes of each predictive baseline variable. Frequencies for each potentially predictive variable and category and for each outcome (successful and unsuccessful), respectively, were set to *n* ≥ 5, and any statistically significant predictive variable in the univariate analysis based on a category with a frequency <5 was not used in the next step of the modelling. In step two, statistically significant predictive variables, based on the univariate analyzes, were included in three multivariate models, one for each outcome, where the RR and 95% CI were estimated.

No intercorrelations between the three outcomes regarding change in outcomes (between start and discharge) did exceed *r*_s_ > 0.4. Hence, all three outcomes were included in the analyzes as independent outcomes.

Statistical analyzes for between-groups comparisons and nonparametric correlations were performed using IBM SPSS Statistics 25, whilst modified Poisson regression was performed using SAS, version Enterprise 8.3 (SAS Institute Inc.). *p* values less than 0.05 were considered statistically significant.

## 3. Results

### 3.1. Effect of Predictive Baseline Variables on Successful Outcomes

Nine of the suggested 17 predictive baseline variables included in the analyzes showed statistically significant prediction on the outcome in the *univariate analyzes* for the three outcomes: “ability to manage overall life circumstances”, EQVAS, and NPRS.

The variables that showed statistically significant RRs in the *univariate analysis* were for the outcomes:

- “ability to manage overall life circumstances”: NPRS and classification of localized/regional/generalized pain
- EQVAS: anxiety/depression, duration, and NPRS
- NPRS: EQVAS, DRI, age, classification of localized/regional/generalized pain, amount of weekly physical exercise, information on if assessed by a team or by solely a physical therapist before start of the program, and duration (see Table 4).

In the *multivariate analysis*, the statistically significant predictive variables from the univariate analyzes were included, and the RR and 95% CI for each of the three outcomes showed the following result:

Patients with pain ratings of NPRS 6–7 or 8–10 at start of rehabilitation were in both cases 14% less likely to improve in “ability to manage overall life circumstances,” as compared to those rating NPRS 0–5 (RR = 0.86; 95% CI 0.77–0.97 and RR = 0.86; 95% CI 0.74–1.00) (see Table 4).

Patients reporting anxiety/depression at start of rehabilitation were 1.48 times more likely to improve in overall health status (EQVAS) as compared to those who did not report anxiety/depression at the baseline (RR = 1.48; 95% CI 1.16–1.88). Likewise, patients rating severe pain (NPRS 8–10) at start of rehabilitation were 1.48 times more likely to improve in overall health status (EQVAS) as compared to patients rating mild pain (NPRS 0–5) (RR = 1.48; 95% CI 1.03–2.15), while patients reporting the shortest pain duration (3 months to 1 year) were 1.61 times more likely to improve in overall health status (EQVAS) as compared to patients reporting pain duration >5 years (RR = 1.61; 95% CI 1.13–2.29).

Patients being diagnosed with regional or generalized pain were 36% less likely to improve in pain rating (NPRS) at discharge as compared to patients diagnosed with localized pain at the baseline (RR = 0.64; 95% CI 0.41–1.00) (see Table 4).

Three potentially predictive baseline variables in the univariate analyzes had a category with a frequency of *n* < 5. Thus, these three results were not used in step two of the analyzes: for the outcome “ability to manage overall life circumstances,” the category BMI and sedentary time were statistically significant but had one category with a frequency of *n* < 5, respectively, and for the outcome EQVAS, the category EQVAS at the baseline had one category with a frequency of *n* < 5. Consequently, these three results were not regarded as statistically significant and thus not used in the multivariate modelling.

## 4. Discussion

The main findings of the present study were that patients rating mild pain at the baseline were more likely to improve in “ability to manage overall life circumstances” as compared to those who rated moderate or severe pain. Further, patients reporting the shortest pain duration were more likely to improve in overall health as compared to those with the longest pain duration. Patients reporting anxiety/depression and patients rating severe pain at the baseline were more likely to improve in overall health status as compared to those with better baseline presentations. Finally, patients diagnosed with localized pain as opposed to regional/generalized pain at the baseline were more likely to rate reduced pain after the rehabilitation program PT-PRP. Our results suggest that for a successful outcome of this and similar kinds of rehabilitation, the patients should probably be offered this rehabilitation at an early stage to lower the risk of prolonged pain and consequently the risk of widespread pain development. Also, the results suggest that patients reporting anxiety/depression and severe pain at the baseline can still improve overall health after this kind of individual, physiotherapist-led rehabilitation of median 9 sessions. The question posed in the title could be replied in the affirmative. But, the results showed no strong and consistent predictive baseline variable, as for example a statistically significant baseline variable for all three outcomes. Therefore, the results found here can contribute to the clinical reasoning that still must be relied on in the clinic when deciding type of rehabilitation for patients with chronic musculoskeletal pain.

To the best of our knowledge, there are no studies on predictive factors in accordance with the ones presented here regarding a patient population with chronic, severe pain who has completed a similar one-to-one, physiotherapist-led rehabilitation program. Various patient-populations with various severeness of symptoms have been studied, various content of rehabilitation programs have been used, and the studies have been performed in various health care settings in specialized and in primary care [20, 23, 40–51]. Moreover, the previous literature shows somewhat conflicting results of that; no strong predictive baseline variables exist to predict successful outcome [20–23, 42, 46, 47], and worse baseline status can be associated with improved outcome after rehabilitation [23, 40, 42, 46–48], but also that poor pretreatment physical and psychological functioning at the baseline can have a negative prognosis [20]. Also, in previous studies, various outcomes and predictive baseline variables are used [20, 23, 40–51]. All these factors reveal the complexity of the research field and make comparisons between studies, the present one included, challenging.

The results of the present study, namely, that no consistent predictive baseline variables can predict successful outcome are, however, supported by studies of multidisciplinary or interdisciplinary pain rehabilitation programs [42, 46, 47]. Likewise, studies of outpatient physiotherapy in patients with mainly localized musculoskeletal pain with shorter duration show similar results [50, 51]. Although the present study comprises a different intervention and group of patients, the fact that no consistent predictive variables were found in the present study adds to previous findings. We therefore recognize that there is still no simple solution on how to assess patients' rehabilitation needs and potential. This makes the clinical question on what intervention should be suggested for which patient, still open. However, our results can be added to the clinical reasoning used in everyday clinical work and described as follows: the complex process comprising a range of skills and strategies using clinical and other data, patient choices, professional judgement, and knowledge to decide on, for example, diagnosis and interventions for each individual patient [52, 53]. We therefore suggest that the results found here can be included as one essential element in this clinical reasoning process.

Results of the present study and of previous research show that worse baseline status can be statistically significantly associated with improvements in outcomes after rehabilitation [23, 40, 42, 46–48]. Higher levels of disability and suffering or poorer mental health at the baseline were shown to display greater improvements [42, 47], and high levels of pain [23], as well as higher degrees of depression and age, yielded a better outcome after multidisciplinary rehabilitation [48]. Although there are differences in populations, outcomes, and predictive variables, the result that worse baseline status can be associated with improvements after rehabilitation is in some agreement with the present study where improved overall health status was associated with baseline reports of anxiety/depression (as opposed to reporting no anxiety/depression) and of severe pain (NPRS 8–10) [42, 47, 54]. Interestingly, previous studies used multidisciplinary rehabilitation, engaging several professionals, while we in the present study used a more defined, physiotherapist-led rehabilitation. The patients included in the present study are not completely comparable to the populations discussed just above. But, our results suggest that patients reporting baseline anxiety/depression or severe pain can still benefit from a more limited intervention as the one studied here and should therefore not be restrained from inclusion in physiotherapist-led interventions.

In a recent systematic review and meta-analysis of 9436 participants, Tseli et al. investigated “prognostic factors for physical functioning” 6 months after multidisciplinary rehabilitation [20]. The authors found that better outcome of function was predicted by better baseline presentations of functioning and of low emotional distress [20]. Even though different predictors and outcomes were used as compared to the present study, there is some accordance with the results of this study, showing that better baseline ratings of localized pain (as opposed to regional/generalized) were related to improved pain intensity after rehabilitation. However, in contrast to our study, these authors concluded that neither pain intensity nor pain duration was associated with their outcome. Several reasons for these differences may exist. For example, the study by Tseli et al. is a systematic review with a much larger sample, and the interventions used were more comprehensive (multidisciplinary pain rehabilitation with an average total of 100 hours) in contrast to the rehabilitation used in the present study with a median of 9 sessions for about 1 hour/session [19, 20]. Also, the cohort in the present study describes another group of patients; a subgroup of patients with CMP still suffers from severe pain but with fewer biopsychosocial consequences. Therefore, they were referred to a physiotherapist and not to multidisciplinary interventions within specialized care. Since the study population and the rehabilitation program used in the present study is not very well investigated, we believe that the results here are of high interest. Also, the results of the present study might be valid for patients with chronic pain in primary care settings. However, professionals in primary care request increased knowledge and skills to fully master the biopsychosocial perspective [55].

Localized pain—as opposed to regional/generalized pain—was in the present study associated with clinically important improvements in pain intensity after rehabilitation. Gerdle et al. found in a registry study of 14,666 participants that widespread pain was associated with longer pain duration and more severe clinical presentations at the baseline but also with a poorer overall treatment outcome after interdisciplinary pain rehabilitation. Therefore, they suggested early interventions for this patient group [56]. Likewise, widespread pain has been found to be associated with poor prognosis, health related quality of life, work disability, and a higher proportion of disability pensions [57, 58]. The statistically significant results of the present study of favorable outcomes associated with mild pain intensity, short duration, and localized pain also point in the direction that these interventions for patients with CMP ought to be offered early, so as not to increase the risk of developing widespread pain and other negative consequences [56]. Therefore, research studying different groups of patients with CMP and how to better tailor program interventions that can be offered early in the course of pain and to many patients with CMP is needed [3, 41].

Some similar individual rehabilitation programs to the one used in the present study have been described previously. For instance, Wippert et al. used graded sensorimotor exercises and behavioral training combined rendering favorable results and recommended that exercise therapy should be individualized as far as possible [59]. Also, Grimby-Ekman et al. emphasized individualization, since they found that improvements in pain intensity were associated with individual-based treatment as compared to group-treatment in multimodal rehabilitations programs [40]. O'Keeffe et al. concluded that disability, but not pain, could be reduced at 6 and 12 months after individualized rehabilitation with cognitive approaches as opposed to group based, multidimensional interventions with the same principles but without the individual approach [16]. However, none of the studies mentioned above investigated a perspective of potentially predictive factors for outcome of individual rehabilitation. We therefore suggest that this kind of inexpensive, individual rehabilitation format where tailored exercises of physical and/or mental training and strategies are used for this subgroup of patients with CMP ought to be studied further.

One limitation of the present study concerns that anxiety/depression was estimated by only one question, although this was from a validated questionnaire (EQ5D). Even if this cannot be compared to more sophisticated instruments, such as hospital anxiety and depression scale (HAD) [60], our results suggest that such a baseline presentation is not a barrier for this kind of physiotherapist-led rehabilitation. It should however be noted, that since it has been shown that there is a bidirectional influence of similar magnitude of pain and mental illness, both conditions must be monitored and met with appropriate, targeted interventions [61].

Another potential limitation is that not all eligible patients could be included in the final study cohort. Some patients in our clinic developed other diseases and needed other types of care, such as surgical interventions. Yet, the main reasons for not including patients in the final cohort were administrative. For example, administrative routines regarding questionnaires changed and questionnaires were exchanged during the five-year period due to developments in clinical work or regulatory changes in care that the unit had to follow. We believe that this is how everyday care works, and importantly, this has not affected our patient composition in the final cohort in any systematic way. For example, when questionnaires have been changed, it has not affected some selected patients but all patients. So, motivational and similar factors were only to a lesser extent reasons for not being included in the final cohort. In fact, only 41 patients (8%) chose to discontinue the program. We did not have access to baseline data for these patients and could therefore not perform a dropout analysis.

Further, the sample can be considered small when the variables were divided into 2–4 categories for each outcome (successful and unsuccessful outcome) in the regression analysis. This led us to collapse categories before starting the regression analysis since we decided in advance, that any variable with a frequency of *n* < 5 for any category should be deleted. Still, only three statistically significant potentially predictive variables had a category with a frequency of n < 5 in the univariate analyzes and were thus not used in the multivariate analyzes. Therefore, we do not believe that this had any major impact on our main conclusions.

We had no access to information on the patients' employment or working status–factors shown to be important in chronic pain [62, 63]. However, we had access to a broad perspective of potentially predictive baseline variables, in several different domains described in the literature, known to be important for predicting outcome after rehabilitation in patients with CMP.

In conclusion, of 17 potentially predictive baseline variables, mild pain ratings, short pain duration, and localized baseline pain were statistically significantly associated with improvements after individual, physiotherapist-led rehabilitation for patients with CMP. This suggests that this type of pain rehabilitation probably should be offered early. Reporting anxiety/depression or severe pain at the baseline did not hinder the improvements of overall health.

## Figures and Tables

**Table 1:** Study population of 274 patients at start of the rehabilitation program PT-PRP.

Age, mean ± SD (min–max) (years)	42 ± 13.4 (18–77)
BMI, mean ± SD (kg/m^2^)	25.0 ± 4.1
Women, *n* (%)	194 (71)
Pain duration, median (q1, q3) (days)	1015 (516, 2514)
Pain distribution as diagnosed by the physiotherapist, *n* (%), valid *n* = 265^†^	
Localized pain	104 (39)
Regional pain	145 (55)
Generalized pain	16 (6)
Main pain location, *n* (%), valid *n* = 270^††^	
Neck pain	76 (28)
Multiple painful areas	65 (24)
Lumbar, thoracic spine, or pelvic pain	60 (22)
Hip, knee, ankle, or foot pain	47 (17)
Shoulder, elbow, or hand pain	22 (8)
NPRS^*a*^, median (q1, q3)	7 (5, 8)
DRI^*b*^, median (q1, q3) (mm)	49 (31, 62)
EQVAS^*c*^, median (q1, q3) (mm)	50 (35, 70)
Physically active^*d*^ minimum 150 min/week according to rating, *n* (%) valid *n* = 134	48 (36)
Number of individual rehabilitation-goals at start^*e*^, median (q1, q3)	2 (2, 3)

^*a*^ = NPRS (numeric pain rating scale) 0–10; 0 = no pain, 10 = worst pain imaginable, 7 = severe pain; ^*b*^ = DRI (disability rating index, mean mm of physical disability of 12 activities) 0–100, 0 = no disability, 100 = cannot at all carry out; ^*c*^ = EQVAS (EuroQol five dimensions questionnaire, visual analogue scale), 0–100, 0 = worst perceived health imaginable 100 = best perceived health imaginable; ^*d*^ = question according to the Swedish National Institute of Public Health; “For how long do you practice leisure exercise per week, for example walking, biking or gardening?” (missing *n* = 134 due to that the question was not added until 2017); ^*e*^ = number of value-oriented, activity-based goals formulated by the patient and the physiotherapist at start of the rehabilitation and likely to be achieved during the program. ^†^ = missing number of patients = 9 due to missing answers from patients in questionnaires for this specific question. ^††^ = missing number of patients = 4 due to missing answers from patients in questionnaires for this specific question. See also methods section. Table 1 is modified from that by Trulsson Schouenborg et al. [19].

**Table 2:** Description of the cohort at the group level at start and results in main outcomes at discharge and at 1-year follow-up, *n* = 274.

Assessment	Start of rehabilitation	Discharge of rehabilitation	Follow-up 1 year after end of rehabilitation
Single question^*a*^: “Has your rehabilitation influenced your ability to manage overall life circumstances?” percent rating “improved” or “much improved”	N.A	85%	74%
			*p*=0.367 as compared to discharge
		*n* = 256	*n* = 187

NPRS^*b*^ md (q1, q3), group level	7 (5, 8)	5 (3, 7)	5 (3, 7)
		*p* < 0.001 as compared to start	*p* < 0.001 as compared to start *p*=0.363 as compared to discharge
	*n* = 265	*n* = 250	*n* = 183

EQVAS^*c*^, md (q1, q3) group level	50 (35, 70)	65 (50, 80)	65 (45, 80)
		*p* < 0.001 as compared to start	*p* < 0.001 as compared to start *p*=0.821 as compared to discharge
	*n* = 259	*n* = 245	*n* = 179

^*a*^ = question used in the Swedish Quality Registry for Pain Rehabilitation, SQRP. ^*b*^ = NPRS (numeric pain rating scale) 0–10; 0 = no pain and 10 = worst pain imaginable. ^*c*^ = EQVAS (EuroQol five dimensions questionnaire, visual analogue scale), 0–100, 0 = worst perceived health imaginable and 100 = best perceived health imaginable. See also methods section.

**Table 3:** Potentially predictive baseline variables and their categorizations.

Potentially predictive variable	Definition of categories
Sex	Man^*∗*^
	Woman

Age (years)	0–40, young^*∗*^
	41–60, middle aged
	61–80, elderly

BMI, body mass index (kg/m^2^)	0–24, under + normal weight∗
	25–29.9, overweight
	>30, obese

Pain duration	3 months–1 year (y)
	1 year–5 years
	>5 years^*∗*^

NPRS^*a*^	0–5 mild pain^*∗*^
	6-7 moderate pain
	8–10 severe pain

DRI^*b*^, mean mm of 12 activities	0–37^*∗*^
	38–57
	58–100

EQVAS^*c*^ (mm)	0–40^*∗*^
	41–70
	71–100

Ability to reduce pain^*d*^, 0–6	0 + 1 + 2^*∗*^
	3 + 4
	5 + 6

Physical exercise^*e*^ (min/week)	0–<30^*∗*^
	30–<90
	90–>120

Leisure exercise^*e*^ (min/week)	0–60^*∗*^
	60–150
	150–>300

Sedentary time per day^*e*^ (hours/day)	10-as good as all day
	4–9 Never-3^*∗*^

Single question on anxiety/depression^*f*^	Reporting NO anxiety/depression^*∗*^
	Reporting anxiety/depression

Disability living allowance	No
	Yes^*∗*^

Assessed by a team or by a physiotherapist before start of the program	Physiotherapist^*∗*^
	Team or a physiotherapist together with other professionals (psychologist and MD)

Classification of pain	Localized pain^*∗*^
	Regional pain/generalized pain

Number of sessions during the rehabilitation period	1–3
	4–7
	8–14
	15–33^*∗*^

Number of different types of interventions	0–2
	3–4
	5–7^*∗*^

^*∗*^indicates the reference category. For categorization, see also methods. ^*a*^ = NPRS (numeric pain rating scale), 0–10; 0 = no pain and 10 = worst pain imaginable; ^*b*^ = DRI (disability rating index, mean mm of physical disability of 12 activities) 0–100, 0 = no disability and 100 = cannot at all carry out [32, 33]; ^*c*^ = EQVAS (EuroQol five dimensions questionnaire, visual analogue scale), 0–100, 0 = worst perceived health imaginable and 100 = best perceived health imaginable; ^*d*^ = single question, ratings 0–6, 0 = cannot reduce pain at all, 6 = high ability to reduce pain; ^*e*^ = amount of weekly physical exercise/leisure exercise/sedentary-time per day [34, 35]; ^*f*^ = anxiety/depression as reported from the single question on anxiety/depression in EQ-5D, 3L, and 5L, categorization according to “reporting NO anxiety/depression” = answer no. 1 + 2 in 3L and in 5L, “reporting anxiety/depression” = answer no. 3 in 3L and no 3 + 4+5 in 5L. See also Methods section. MD = medical doctor.

**Table 4:** Univariate and multivariate analyses for three outcomes.

Predictive variable	Categories	Univariate analysis	Multivariate analysis
		RR (95% CI)	RR (95% CI)
*Outcome: “improved” or “much improved” ability to manage overall life circumstances at discharge*			
NPRS^*a*^	NPRS 0–5, mild pain	1.00 ref	1.00 ref
	NPRS 6-7, moderate pain	**0.88 (0.79–0.98)**	**0.86 (0.77–0.97)**
	NPRS 8–10, severe pain	0.90 (0.80–1.03)	**0.86 (0.74–1.00)**
Pain distribution	Localized pain	1.0 ref	1.0 ref
	Regional/generalized pain	**0.91 (0.82–1.00)**	0.95 (0.85–1.06)

*Outcome: improved perceived overall health status, EQVAS, from start to discharge*			
Anxiety/depression^*b*^	Reporting NO anxiety/depression	1.0 ref	1.0 ref
	Reporting anxiety/depression	**1.65 (1.29–2.10)**	**1.48 (1.16–1.88)**
Duration	3 months–1 year	**1.66 (1.14–2.42)**	**1.61 (1.13–2.29)**
	1–5 years	1.34 (0.93–1.92)	1.17 (0.82–1.65)
	>5 years	1.0 ref	1.0 ref
NPRS	NPRS 0–5, mild pain	1.00 ref	1.00 ref
	NPRS 6-7, moderate pain	1.19 (0.83–1.71)	1.25 (0.87–1.79)
	NPRS 8–10, severe pain	**1.53 (1.06–2.21)**	**1.48 (1.03–2.15)**

*Outcome: improved pain rating, NPRS, from start to discharge*			
EQVAS^*c*^	0–40 mm	1.00 ref	1.00 ref
	41–70 mm	1.33 (0.92–1.91)	1.53 (0.86–2.72)
	71–100 mm	**1.95 (1.33–2.86)**	2.46 (0.95–6.37)
DRI^*d*^	0–37 mm	1.00 ref	1.00 ref
	38–57 mm	0.82 (0.59–1.13)	1.32 (0.81–2.16)
	58–100 mm	**0.66 (0.46–0.95)**	0.94 (0.46–1.91)
Age	0–40 years	1.00 ref	1.00 ref
	41–60 years	1.19 (0.88–1.60)	1.07 (0.66–1.73)
	61–80 years	**1.57 (1.05–2.34)**	1.80 (0.95–3.40)
Pain distribution	Localized pain	1.00 ref	1.00 ref
	Regional/generalized pain	**0.58 (0.44–0.76)**	**0.64 (0.41–1.00)**
Amount of weekly physical exercise^*e*^	0–<30 min	1.00 ref	1.00 ref
	30 min–90 min	1.16 (0.70–1.92)	0.79 (0.40–1.58)
	90–>120 min	**1.62 (1.07–2.43)**	1.40 (0.90–2.19)
Assessed by a team or by a physical therapist before start	Assessment by a physical therapist	1.00 ref	1.00 ref
	Assessment by a team	**0.60 (0.46–0.80)**	0.79 (0.51–1.23)
Duration	3 months–1 year	**1.58 (1.06–2.34)**	0.94 (0.49–1.80)
	1–5 years	1.27 (0.87–1.84)	0.65 (0.39–1.06)
	>5 years	1.00 ref	1.00 ref

Statistically significant results are given in bold. The three outcomes are manage overall life circumstances, ratings of perceived overall health status (EQVAS), and ratings of pain (NPRS). For categories, see Table 3. ^*a*^ = NPRS (numeric pain rating scale) 0–10; 0 = no pain and 10 = worst pain imaginable. ^*b*^ = Anxiety/depression as reported from the single question on anxiety/depression in EQ-5D, 3L, and 5L, categorization according to “reporting NO anxiety/depression” = answer no. 1 + 2 in 3L and in 5L, “reporting anxiety/depression” = answer no. 3 in 3L and no 3 + 4+5 in 5L. ^*c*^ = EQVAS (EuroQol five dimensions questionnaire, visual analogue scale), 0–100, 0 = worst perceived health imaginable and 100 = best perceived health imaginable. ^*d*^ = DRI (disability rating index, mean mm of physical disability of 12 activities) 0–100, 0 = no disability and 100 = cannot at all carry out. ^*e*^ = Amount of weekly physical exercise. For references, see Table 3.

## Data Availability

The data that support the findings of this study are available from Region Skåne but restrictions apply to the availability of these data since they are based on medical records, which were used under license for the current study and so are not publicly available. Data are however available from the corresponding author upon reasonable request and with permission of “The Commission of quality registry for caring-databases at Region Skåne” (KVB, kunskapsstyrning@skane.se, after approval of “Application for disclosure of personal information from the Region Skåne databases and quality registries”) and after approval of the Swedish Ethical Review Agency, Etikprövningsmyndigheten at https://etikprovningsmyndigheten.se/en/. Anonymized data can be available upon reasonable request to the corresponding author after relevant ethical approvals that apply.

## Abbreviations

CMP: Chronic musculoskeletal pain
RR: Risk ratio
CI: Confidence interval
PT-PRP: Physiotherapy pain rehabilitation program
VAS: Visual analogue scale
NPRS: Numeric pain rating scale
DRI: Disability rating index
EQ-5D and EQVAS: EuroQol five dimensions questionnaire
BMI: Body mass index
MCID: Minimal clinical important differences
HAD: Hospital anxiety and depression scale.
